# Literary Notes

**Published:** 1901-11

**Authors:** 


### LITERARY NOTES.

Messrs. Battle & Co., St. Louis, have issued chart No. 5 of the series, illustrating the fractures of the long bones. This chart delineates fracture of fibula, with dislocation of foot outward,—Pott's fracture.

The Enno Sander prize has for 1901-1902, been generously increased by its founder to consist of a gold medal, valued at $100 and $100 in cash. The subject for this year is, The most practicable organisation for the medical department of the United States army in active service. The conditions are that it is open to competition by all persons eligible to active or associate membership in the association of military surgeons of the United States; that essays must consist of not less than 10,000 nor more than 20,000 words, and that they must reach the secretary of that association, Carlisle, Pa., not later than February 28, 1902.

The Nicholas Senn prize medal will be conferred upon that member of the American Medical Association who shall present on or before March 1, 1902, the best essay on some surgical subject. Communications may be addressed to any member of the committee, consisting of the following: Dr. Herbert L. Burrell, 22 Newbury Street, Boston, Mass.; Dr. Edward Martin, 415 S. 15th Street, Philadelphia, Pa.; Dr. Charles H. Mayo, Rochester, Minn.

